# Transcription factor-induced activation of cardiac gene expression in human c-kit+ cardiac progenitor cells

**DOI:** 10.1371/journal.pone.0174242

**Published:** 2017-03-29

**Authors:** Tareq Al-Maqtari, Kyung U. Hong, Bathri N. Vajravelu, Afsoon Moktar, Pengxiao Cao, Joseph B. Moore, Roberto Bolli

**Affiliations:** Institute of Molecular Cardiology, Department of Medicine, University of Louisville, Louisville, KY, United States of America; Centro Cardiologico Monzino, ITALY

## Abstract

Although transplantation of c-kit+ cardiac progenitor cells (CPCs) significantly alleviates post-myocardial infarction left ventricular dysfunction, generation of cardiomyocytes by exogenous CPCs in the recipient heart has often been limited. Inducing robust differentiation would be necessary for improving the efficacy of the regenerative cardiac cell therapy. We assessed the hypothesis that differentiation of human c-kit+ CPCs can be enhanced by priming them with cardiac transcription factors (TFs). We introduced five different TFs (*Gata4*, *MEF2C*, *NKX2*.*5*, *TBX5*, and *BAF60C*) into CPCs, either alone or in combination, and then examined the expression of marker genes associated with the major cardiac cell types using quantitative RT-PCR. When introduced individually, *Gata4* and *TBX5* induced a subset of myocyte markers. Moreover, *Gata4* alone significantly induced smooth muscle cell and fibroblast markers. Interestingly, these gene expression changes brought by *Gata4* were also accompanied by morphological changes. In contrast, *MEF2C* and *NKX2*.*5* were largely ineffective in initiating cardiac gene expression in CPCs. Surprisingly, introduction of multiple TFs in different combinations mostly failed to act synergistically. Likewise, addition of *BAF60C* to *Gata4* and/or *TBX5* did not further potentiate their effects on cardiac gene expression. Based on our results, it appears that *GATA4* is able to potentiate gene expression programs associated with multiple cardiovascular lineages in CPCs, suggesting that *GATA4* may be effective in priming CPCs for enhanced differentiation in the setting of stem cell therapy.

## Introduction

In contrast to the long-standing belief that the mammalian heart is a post-mitotic or terminally differentiated organ, previous reports have demonstrated that the adult mammalian heart possesses a capacity of cardiomyocyte renewal [1–5]. Beltrami and colleagues first described a unique resident cardiac cell population with characteristics of stem cells in the rat heart [6]. This population of cells was found to be positive for c-kit (c-kit+), a receptor tyrosine kinase, and when isolated and grown in culture, they were self-renewing, clonogenic, and multipotent, being able to differentiate into cardiomyocytes, smooth muscle, and endothelial cells. Since then, c-kit+ CPCs have been described in multiple mammalian species, including human [7–11]. Also, discovery of specialized niches within the heart which contain clusters of undifferentiated c-kit+ CPCs and early-lineage committed cells (i.e., c-kit and GATA4, MEF2C, or Ets1 double-positive cells) strongly suggests that they not only reside stably in the heart but also are specifically “programmed” to give rise to multiple cardiac cell types [9]. Moreover, when injected into an ischemic heart, they reconstitute differentiated myocardium with new vessels and myocytes [6]. In a recent phase I clinical trial, c-kit+ CPCs isolated from patients with ischemic cardiomyopathy have been shown to significantly improve heart function and the quality of life when transplanted back into the patients via intracoronary injection [11, 12], clearly demonstrating the utility of these cells in developing stem cell therapies for the treatment of ischemic cardiomyopathy.

However, current cell therapy with adult c-kit+ CPCs for ischemic cardiomyopathy is largely limited by the poor survival and retention of transplanted stem cells [13, 14] and also by the lack of robust *de novo* differentiation of transplanted stem cells into mature cardiac cell types [14, 15]. Although methods of enhancing the viability of CPCs following transplantation have been previously explored [16, 17], so far no study has tested whether or not promoting the cardiovascular differentiation of CPCs can further enhance the efficacy of the cardiac progenitor cell therapy. One of the innovative methods recently employed to direct differentiation of stem/progenitor cells is to introduce tissue- or cell type-specific transcription factors (TFs), a method often referred to as ‘forward programming.’ For instance, Takeuchi and Bruneau have shown that extra-cardiac mesoderm in the mouse embryo can be programmed into cardiac tissue by introducing four cardiac TFs, *Gata4*, *Nkx2*.*5*, *Tbx5*, and *Baf60c* [18]. Also, differentiation of human embryonic stem (ES) cells into cardiomyogenic lineage can be directed by introducing *GATA4*, *NKX2*.5, *TBX5*, and *BAF60C* [19]. A similar study has reported that the combination of *GATA4*, *BAF60C*, and *MESP1* was most effective for cardiac forward programming of both human induced pluripotent stem cells and ES cells [20]. *Gata4*, *Mef2c*, and *Tbx5* were sufficient to even reprogram cardiac and tail-tip fibroblasts into functional cardiomyocytes [21], although this idea has been recently challenged [22]. Taken together, these studies demonstrate that cardiac TF-driven reprogramming is not only a feasible but also a powerful approach in directing cardiogenic differentiation of different cell populations.

In the present study, we examined the effects of overexpressing five cardiac TFs (*Gata4*, *MEF2C*, *NKX2*.*5 TBX5*, and *BAF60C*) on the differentiation state of c-kit+ human CPCs in culture. After introducing the TFs either individually or in combination, expression of marker genes associated with major cardiac cell types were assessed by quantitative RT-PCR, immunofluorescence, and Western blot. The results show that among the TFs tested, Gata4 is effective in initiating expression of genes of multiple lineages when overexpressed in c-kit+ CPCs. Surprisingly, however, combinations of TFs were largely ineffective in potentiating the cardiac gene expression programs in CPCs, demonstrating the complexity of the cardiac gene regulatory network.

## Materials and methods

### Isolation and culture of c-kit+ cardiac progenitor cells

c-Kit+ human cardiac progenitor cells were isolated and cultured as previously described [23]. A written consent agreement was obtained for collection of discarded right atrial appendages according to a protocol approved by the Institutional Review Boards on human subject research at University of Louisville. Four different patient cell populations were used for the present study. The only medical history that we could obtain at the time was that the cardiac tissues were from non-diabetic patients. Briefly, human atrial appendages were enzymatically digested [23], and the resulting cell suspension was cultured in Ham's F12 medium containing 10% FBS, 10 ng/ml human bFGF (PeproTech), 0.2 mM L-glutathione (Sigma), and 0.005 U/ml human erythropoietin (Sigma). The adherent cells were continuously cultured with medium change every other day or subcultured every 4–5 days. The c-kit+ CPCs were enriched using the c-kit MACS kit according to the manufacturer's instructions (Miltenyl Biotec). Briefly, total populations of adherent cells between P1 to P3 were collected and incubated with microbeads directly conjugated to mouse monoclonal anti-human c-kit antibody (clone AC126) at 4°C to 8°C for 20 min. The cells were then loaded into a MACS column which was placed in the magnetic field of MACS Separator (Miltenyi Biotec). The labeled c-kit+ cells were retained on the column and unlabeled cells were eluted. After column was removed from the magnetic field, the magnetically retained c-kit+ cells were collected as positively selected cell fraction for further expansion. To exclude the potential contamination of lin+ cells (such as T cells, B cells, NK cells, dendritic cells, monocytes, granulocytes, erythroid cells, and their committed precursors), a Lineage Cell Depletion Kit (Miltenyl Biotec) was used to purify c-kit+ cell populations as previously described [23]. The magnetically labeled Lin+ cells were depleted by retaining them on a MACS column in a magnetic field while unlabeled lin− cells passed through the column and were collected for further expansion and/or analysis.

### Lentivirus expressing cardiac transcription factors

Lentivirus expressing the cardiac transcription factors used in the current study was produced using ViraPower^TM^ Lentiviral Expression System (Invitrogen) according to manufacturer’s instructions. The following MGC verified full-length cDNA clones for the transcription factors were purchased from OpenBiosystems: human NKX2.5 cDNA (MHS1010-7430146; Clone ID 5225103; NCBI Accession: BC025711), human MEF2C cDNA (MHS1010-7295133; Clone ID: 4815933; NCBI Accession: BC026341), human TBX5 cDNA (MHS1010-7430001; Clone ID: 5204163; NCBI Accession: BC027942), and human BAF60C cDNA (IMAGE:3608512; NCBI Accession: BC002628). A retroviral expression construct for mouse Gata4 (NCBI Accession: NM_008092.3) was a kind gift from Dr. Deepak Srivastava (UC San Francisco). The coding sequences for each transcription factor or mCherry were PCR-amplified using Pfu HF polymerase (Agilent) and subcloned into pLenti6/V5-D-TOPO vector (Invitrogen) according to the manufacturer’s instructions. Primers used for the PCR were the following: 5’-CACCATGTACCAAAGCCTGGCCATG-3’ and 5’-CGCGGTGATTATGTCCCCATGA-3’ for Gata4; 5’-CACCATGTTCCCCAGCCCT-3’ and 5’-CCAGGCTCGGATACCATGC-3’ for NKX2.5; 5’-CACCATGGGGAGAAAAAAGATTCA-3’ and 5’-TGTTGCCCATCATTCAGAAAGTC-3’ for MEF2C; 5’-CACCATGGCCGACGCAG-3’ and 5’-GCTATTGTCGCTCCACTCTGGC-3’ for TBX5; and 5’-CACCATGGTGAGCAAGGGC-3’ and 5’-CTACTTGTACAGCTCGTCCATGCC-3’ for mCherry. For generation of pLenti6-mCherry expression construct, pmCherry-C2 vector (K. U. Hong) was used as the PCR template. For generation of 3xFLAG constructs, the following oligos were synthesized, annealed and inserted into the BamHI site of pLenti6/V5-TOPO vector: 5’-GATCCACCATGGATTACAAGGATGACGACGATAAGGATTACAAGGATGACGACGATAAGGATTACAAGGATGACGACGATAAGGGG-3’ and 5’-GATCCCCCTTATCGTCGTCATCCTTGTAATCCTTATCGTCGTCATCCTTGTAATCCTTATCGTCGTCATCCTTGTAATCCATGGTG-3’. Human BAF60C coding sequences were PCR amplified using the following primers and cloned into EcoRI and SalI sites of pLenti6-3xFLAG vector from above: 5’-TTTGAATTCGATGACTCCAGGTCTTCAGCA-3’ and 5’-AAAGTCGACCTAGGTGTTGCGCACAACC-3’

Each batch of virus was concentrated 10 or 100 times using Lenti-X Concentrator (Clontech) according to the manufacturer’s instructions and resuspended in complete CPC media. Aliquots were made and stored at -80°C until use. Virus titers were determined by qPCR-based measurement of integrated copies of viral genome following transduction of CPCs with varying dilutions of each virus. For calculation of the copy numbers of virus genome integrated into the host, serial dilutions of pLenti6 vector were used to generate the standard curve. Briefly, CPCs transduced with varying dilutions of virus were harvested after 4 days. Genomic DNA was isolated, and 50 ng of DNA was analyzed by qPCR as described below. The following primers were used for the qPCR analysis: 5’-GCTCAGTTCCAGTTGCTTG-3’ and 5’-GCAGTGAGCCAAGATTGCAC-3’ for human HLA-A (for human/CPC genomic DNA) [13]; and 5’-CATCTTGAGCCCCTGCGGACG-3’ and 5’-CCGTCGGCTGTCCATCACTGTC-3’ for integrated lentiviral vector. For the assay, mCherry virus served as a reference. The efficiency of transduction with each dilution of mCherry virus was assessed by measuring the percentage of mCherry-positive cells, and it was plotted against the number of viral genomes integrated into CPCs to obtain a standard curve. Based on the curve, the volume of virus required to achieve 70–80% transduction efficiency was calculated for each virus batch.

### Lentivirus transduction of CPCs

CPCs were plated on 12-well plates the day before transduction at a density of approximately 1.0 x 10^5^ cells per well. Next day, the media was replaced with 250 μl of complete media containing appropriate dilution of virus and 6 μg/ml Polybrene (Sigma), and on the following day, the media was replaced with fresh complete media. The cells then remained on the same plate until harvest at specified time points (i.e., 7, 10, or 14 days post-transduction). The media was replaced every 3 days. Each treatment was done in quadruplicate.

### RNA isolation and quantitative RT-PCR

Total RNA was isolated from CPCs using RNeasy Mini Kit (with DNase treatment) (Qiagen) according to the manufacturer’s instructions. cDNA was synthesized using 250 ng of total RNA using AffinityScript Multiple Temperature cDNA Synthesis Kit (Agilent) according to the manufacturer’s instructions. Samples were analyzed for mRNA levels of indicated markers using SYBR Green Master Mix (Applied Biosystems) and 7900HT Fast Real Time PCR System with SDS version 2.4.1 (Applied Biosystems). Each gene-specific primer set was initially validated based on the product size, and each PCR product was then sequence-verified (data not shown). GAPDH mRNA was used as an internal (house-keeping gene) control and the relative expression of each transcript was calculated using 2^-ΔΔCt^ method. The final data represents the average of four biological replicates. Sequences of the primers used in the present study are listed in Table 1. Dunnett’s test was used to test the statistical significance of the relative fold changes in the gene expression compared to the control.

**Table 1:** List of primers used for qPCR analysis.

*Target Gene*	*Gene Symbol*		*Primer Sequences (5' to 3')*	*Product Size (bp)*
**ACTN2**	*ACTN2*	*forward*	CTGCCGAGCAGGTCATCGCC	210
		*reverse*	AGCATCACAGATCGCTCTCCCCG	
**α-Actinin-2**	*ACTC*	*forward*	CATGGGGCAGGCTGGTAGGTG	487
		*reverse*	CCGACCCAGAGTCAGGGATCAAAA	
**αMHC**	*MYH6*	*forward*	AGGGATAACCAGGGGAAGCACC	339
		*reverse*	AGGTTGAAAAGCACCGCGGG	
**αSMA**	*ACTA2*	*forward*	AGCGACCCTAAAGCTTCCCAGACT	205
		*reverse*	CGGGGGCTGTTAGGACCTTCCC	
**ANP**	*NPPA*	*forward*	GGTCGTGCCCCCACAAGTGC	100
		*reverse*	TGGGCTGACTTCCCCGGTCC	
**BAF60C**	*SMARCD3*	*forward*	TGTCCAGGCCCTGTGGCAGT	151
		*reverse*	TAGCAGGGCTGTGAGGCGCT	
**α-Actin**	*ACTB*	*forward*	GCAGTCGGTTGGAGCGAGCA	197
		*reverse*	ATCACCTCCCCTGTGTGGACTTGG	
**β-MHC**	*MYH7*	*forward*	AGGCCTTGGCCCCTTTCCTCAT	142
		*reverse*	CCTGGTCTGCGCTTCTAGCCG	
**BNP**	*NPPB*	*forward*	CCCCGGTTCAGCCTCGGACT	172
		*reverse*	ACGGATGCCCTCGGTGGCTA	
**Calponin-1**	*CNN1*	*forward*	AACAGCGCCCAAAGGACGCA	199
		*reverse*	CGCTGCAAACCAAACCGCGT	
**CD31**	*PECAM*	*forward*	CAGGCTCCCACTGGCCTGACT	184
		*reverse*	TGCCCTTGCGGTGTTAGGCA	
**c-kit**	*KIT*	*forward*	TGGGCCACCGTTTGGAAAGCT	156
		*reverse*	AGGGTGTGGGGATGGATTTGCTCT	
**Connexin 40**	*GJA5*	*forward*	AGCAAAAAGCGTGGGCAGTTGGA	236
		*reverse*	TGCCCAGCACGAGCATACGG	
**Connexin 43**	*GJA1*	*forward*	CGACCAGTGGTGCGCTGAGC	226
		*reverse*	CCCGCCTGCCCCATTCGATT	
**DDR2**	*DDR2*	*forward*	CTTTGGCTGGACTCTCCTGGCTC	470
		*reverse*	TCCCATGACGGTTCCGCCAAGA	
**FLT1**	*FLT1*	*forward*	TGCGAGCTCCGGCTTTCAGG	182
		*reverse*	TTCTCGCTGCCAGGTCCCGA	
**FSP-1**	*S100A4*	*forward*	TGGTTTGATCCTGACTGCTGTCATG	145
		*reverse*	CTCCCGGGTCAGCAGCTCCT	
**GAPDH**	*GAPDH*	*forward*	GGTGAAGCAGGCGTCGGAGG	127
		*reverse*	GAGGGCAATGCCAGCCCCAG	
**GATA4**	*GATA4*	*forward*	CGGCGAGGAGGAAGGAGCCA	144
		*reverse*	TGGGGGCAGAAGACGGAGGG	
**GATA4 set 2**	*GATA4*	*forward*	CCTCTCCTGTGCCAACTGCCAGA	132
		*reverse*	TTCCGCATTGCAAGAGGCCTG	
**KDR**	*KDR*	*forward*	AGCTCAAGGCTCCCTGCCGT	211
		*reverse*	GCGGGGTGAGAGTGGGTTGG	
**MEF2C**	*MEF2C*	*forward*	GGACAACAAAGCCCTCAGCAGGT	493
		*reverse*	CCGCCAGCGCTCTTCACCTT	
**MEF2C set 2**	*MEF2C*	*forward*	TTGTCCATGTCGGTGCTGGCAT	354
		*reverse*	CGTCCGGCGAAGGTCTGGTG	
**MLC-2V**	*MLY2*	*forward*	CTAGGAGGGGGCTCGCTGCT	180
		*reverse*	TGTGCGGCCACGAAGTACCC	
**Myocardin**	*MYOCD*	*forward*	GTGCCGGGGGAAACCCTTGT	340
		*reverse*	GAAGCCGAGGGCTTGGTGAGG	
**Nkx2.5**	*NKX2-5*	*forward*	CACCGGCCAAGTGTGCGTCT	117
		*reverse*	GCAGCGCGCACAGCTCTTTC	
**SM-MHC**	*MYH11*	*forward*	ACGGGAGAGCTGGAAAAGCAGC	196
		*reverse*	TGGCTTGGCGAATTGCCCGT	
**Smoothelin**	*SMTN*	*forward*	GATGCTGGTGGACTGTGTGCCC	127
		*reverse*	CAGTTCGTGGCGTCGCAGGT	
**Tbx5**	*TBX5*	*forward*	AGTCCCCCGGAACAACTCGAT	235
		*reverse*	ACAGCAGCTGCACCGTCACC	
**Tbx5 set 2**	*TBX5*	*forward*	CCTATCAGTGCCGGGTCTGCG	135
		*reverse*	CTCCCAGCTGTGGGGAGCCA	
**TEAD1**	*TEAD1*	*forward*	TGGAGCCCCGACATCGAGCA	351
		*reverse*	TGGGAAGGTCGGGCGTGGAA	
**Thy1**	*THY1*	*forward*	CCCAGGAGCCGGACACTTCTCA	252
		*reverse*	GGTGGCGTTCCCCAGCCTCA	
**Troponin T**	*TNNT2*	*forward*	AGAAGGCCAAGGAGCTGTGGCA	170
		*reverse*	CCAGCGCCCGGTGACTTTAGC	
**VE-Cadherin**	*CDH5*	*forward*	ACAGCATCTTGCGGGGCGAC	178
		*reverse*	CCCGCGGGAGGGCTCATGTA	
**VEGF**	*VEGFA*	*forward*	TGGCAGATGTCCCGGCGAAG	118
		*reverse*	TAGGCTGCACCCCAGGAAGGG	
**Vimentin**	*VIM*	*forward*	CCGGAGACAGGTGCAGTCCCT	147
		*reverse*	TCATCCTGCAGGCGGCCAAT	
**vWF**	*VWF*	*forward*	ACCCCTGCCCCCTGGGTTAC	322
		*reverse*	TGCAGCCTGGCAGTGATGTCG	

### Immunofluorescence staining

Cells grown on culture plates or glass coverslips were fixed in 3.7% formaldehyde in PBS for 15 min at room temperature and permeabilized using 0.25% Triton X-100 in PBS for 10 min. Following incubation in the blocking solution (5% bovine serum albumin in PBS) for 30 min, the cells were incubated in primary antibody solution (diluted 1:250 in the blocking solution) for 1 h at room temperature and washed twice in PBS. They were then incubated in a solution of secondary antibodies conjugated to fluorochromes (diluted 1:1000 in the blocking solution) for 1 h at room temperature and washed twice in PBS. They were finally counterstained with DAPI (4’,6’-diamidino-2-phenylindole) and mounted on glass slides using Fluoromount (Sigma). Fluorescence images were viewed and acquired using EVOS® FL Cell Imaging System (Life Technologies).

### Antibodies

The antibodies used for the current study are listed below. KDR/VEFGR2 (mouse monoclonal; ab9530; Abcam); α-SMA (mouse monoclonal; A5228; Sigma); troponin T (mouse monoclonal; clone 13–11; Thermo Fisher); α-sarcomeric actinin (mouse monoclonal; A7811; Sigma); FLAG tag (mouse monoclonal; F-tag-01; Applied Biological Materials); Thy1/CD90 (Mouse monoclonal; clone 5E10; BD Pharmingen); SM-MHC (rabbit polyclonal; Abcam); ANP (mouse monoclonal; clone 23/1; Santa Cruz); BNP (mouse monoclonal; clone 50E1; Thermo Fisher); c-kit (rabbit monoclonal; clone YR145; Epitomics); α-tubulin (mouse monoclonal; clone DM1A; Sigma); V5 tag (mouse monoclonal; clone E10; Applied Biological Materials). For staining, the antibodies were diluted 1:100–1:250, and for Western blots, antibodies were diluted 1:500–1:1000 as recommended by the manufacturers.

## Results

### Introduction of cardiac TFs into c-kit+ CPCs

**Fig 1:**
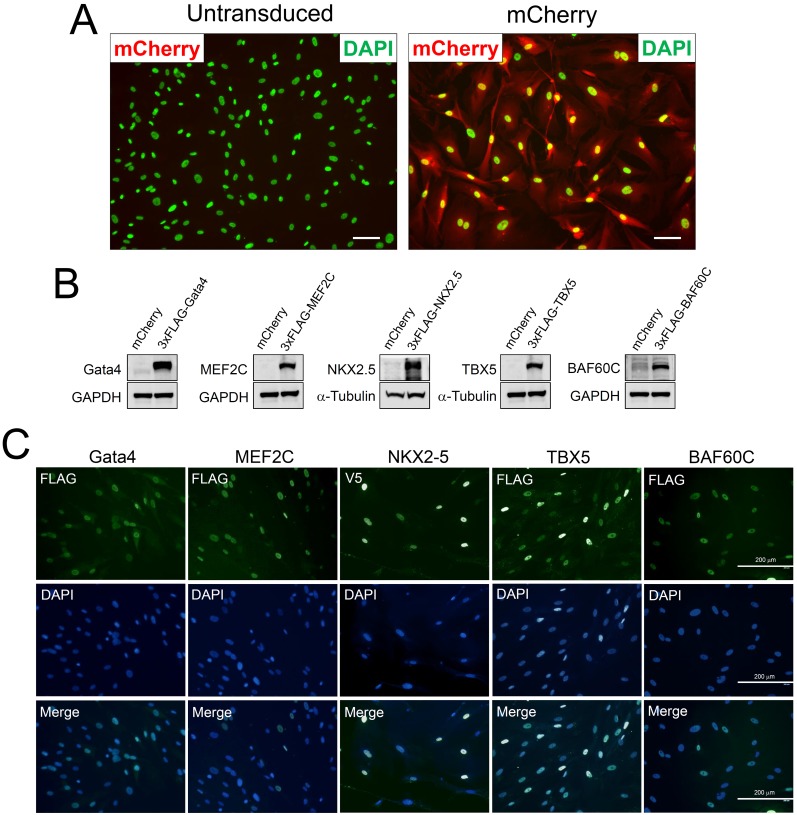
Lentivirus-mediated delivery of transcription factors to CPCs. A, Untranduced or mCherry virus-transduced CPCs were imaged at 4 days post-transduction. Fluorescence of mCherry protein is visible in red. DAPI staining of nuclei was pseudo-colored in green. B, Cells transduced with virus expressing mCherry (control), 3xFLAG-tagged Gata4, MEF2C, TBX5 or BAF60C, or V5-tagged NKX2.5 were assayed by Western blot using antibody against each TF. C, Cells transduced with virus expressing 3xFLAG-tagged Gata4, MEF2C, TBX5 or BAF60C, or V5-tagged NKX2.5 were stained for the indicated epitope (i.e., FLAG or V5) which is shown in monochrome. DAPI images are shown in lower panels. Note that exogenous, epitope-tagged transcription factors localize to the nucleus as expected.

We tested if differentiation of CPCs can be induced or enhanced by ‘priming’ or programming them with TFs. First, we generated lentiviruses expressing four different cardiac transcription factors, *Gata4*, *MEF2C*, *NKX2*.*5*, and *TBX5*, which either alone or in combination have been shown to direct cardiac differentiation of different cell populations or tissues, including ES cells, extra cardiac mesoderm, and fibroblasts [18–21, 24, 25]. TFs were either FLAG-tagged at the N-terminus (Gata4, MEF2C, NKX2.5, and TBX5) or V5-tagged at the C-terminus (NKX2.5) for ease of detection. As shown in Fig 1, we were able to achieve a relatively high efficiency of lentiviral transduction in CPCs which ranged between 70 and 90% (Fig 1A). We then transduced c-kit+ CPCs with virus encoding *Gata4*, *MEF2C*, *NKX2*.*5*, or *TBX5* and verified their expression by Western blot analysis (Fig 1B). Also immunofluorescence staining of transduced cells showed that the exogenous TFs were localized to the nucleus as expected (Fig 1C).

### Overexpression of individual TFs in CPCs

**Fig 2:**
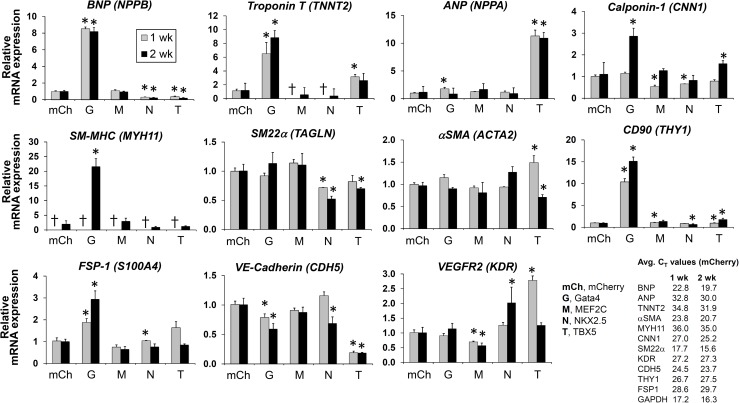
Effect of individual transcription factors on expression of cardiac differentiation markers in human CPCs. Transcription factors (*Gata4* [G], *MEF2C* [M], *NKX2*.*5* [N], and *TBX5* [T]) were introduced to c-kit+ and lin- human CPCs via lentivirus-mediated gene delivery. Cells were then cultured for 1 or 2 weeks. At each time point, cells were harvested, and relative changes in mRNA levels of indicated genes were measured using quantitative RT-PCR. The level of indicated transcript in each group was compared to that of mCherry (mCh)-expressing control group, and is expressed as a relative fold change. For each condition, *n* = 4. Bar graphs show mean ± SEM. *, *p* < 0.05. †, not detected.

**Fig 3:**
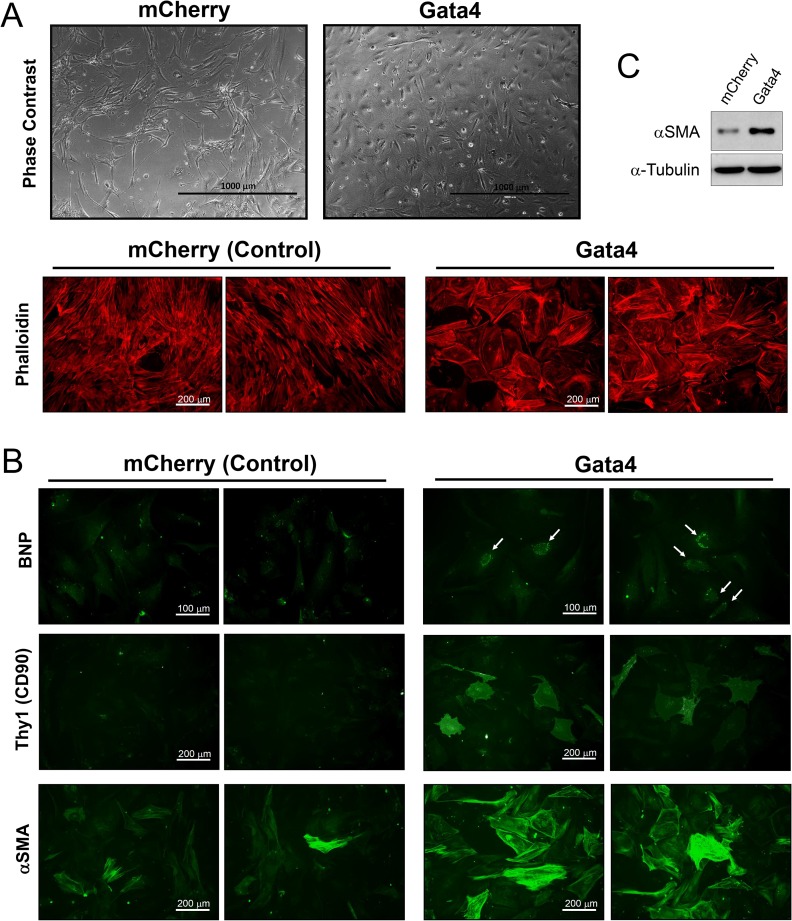
Gata4-induced changes in gene expression and morphology. A, Representative phase-contrast images of mCherry (control) and *Gata4*-overexpressing CPCs are shown (upper panels). The indicated cells were stained with Alexa Fluor 488-conjugated phalloidin after 2 weeks of culture to visualize actin cytoskeleton (lower panels). Note that control cells remain spindle-shaped and elongated, while cells overexpressing Gata4 appear polygonal in their morphology. B, CPCs were transduced as in Panel A and stained for the indicated markers after 2 weeks of culture. BNP staining within *Gata4*-expressing cells are indicated by the arrows. Representative monochromatic micrographs are shown here. C, Cells transduced with virus expressing mCherry (control) or 3xFLAG-tagged Gata4 were harvested 2 weeks later and assayed by Western blot using antibodies against alpha-smooth muscle actin (αSMA) and alpha-tubulin (α-Tubulin; loading control).

First, we introduced the TFs individually into CPCs to see if any of them alone would be sufficient to induce expression of markers associated with cardiac differentiation. CPCs transduced with mCherry virus served as controls. The cells were then cultured for 1 or 2 weeks and analyzed using quantitative RT-PCR to detect changes in the transcript level of more than 30 different cardiac cell type-specific markers (see Table 2). Of note, endogenous transcripts of *GATA4*, *MEF2C*, and *TBX5* were detectable in untreated CPCs, whereas *NKX2*.*5* expression was absent (data not shown). Overexpression of *Gata4* resulted in a significant induction of some of the cardiomyocyte marker mRNAs, including brain natriuretic peptide (BNP; *NPPB*), troponin T (*TNNT2*), and connexin 40 (*GJA5*), within 1 week of expression (Fig 2). However, *Gata4* expression was not sufficient to initiate expression of other cardiomyocyte markers, such as α- and β-MHC and cardiac actin (*ACTC*), in CPCs within the experimental period (data not shown). *Gata4* also induced expression of markers associated with other cardiac cell types. For instance, *Gata4* expression led to increases in the transcript levels of smooth muscle cell markers, including calponin-1 (*CNN1*) and smooth muscle myosin heavy chain (*MYH11*) which became evident by the second week of culture (Fig 2). In addition, two fibroblast markers, such as THY1/CD90 and fibroblast-specific protein 1 (FSP1; *S100A4*), were significantly upregulated following *Gata4* expression in CPCs (Fig 2). However, no significant induction of endothelial cell markers was detected in *Gata4*-expressing cells (Fig 2). Rather, *Gata4* overexpression resulted in marginal suppression of VE-Cadherin (*CDH5*), an endothelial cell marker, expression. Interestingly, introduction of *Gata4* was followed by readily observable changes in cell morphology. While mCherry control cells resumed an elongated and spindle-shaped morphology, *Gata4*-expressing cells became polygonal, often exhibiting prominent stress fibers (Fig 3A).

**Table 2:** Markers of cardiac differentiation examined by quantitative RT-PCR.

	Endothelial	Smooth	Transcription	Mesenchymal/		Loading
Myocyte	Cell	Muscle Cell	Factors	Fibroblast	Stem Cell	Control
***ANP (NPPA)***	***KDR/FLK1***	***αSMA (ACTA2)***	***GATA4***	***THY1/CD90***	***c-kit (KIT)***	***GAPDH***
***BNP (NPPB)***	***vWF***	***SM22α (TAGLN)***	***NKX2*.*5***	***FSP1 (S100A4)***		***β-Actin (ACTB)***
***α-MHC (MYH6)***	***CD31 (PECAM1)***	***SM-MHC (MYH11)***	***MEF2C***	***DDR2***		
***β-MHC (MYH7)***	***VE Cadherin (CDH5)***	***Calponin-1 (CNN1)***	***TBX5***	***Vimentin (VIM)***		
***α-Actinin2 (ACTN2)***	***VEGF (VEGFA)***	***Smoothelin (SMTN)***	***BAF60C (SMARCD3)***			
***Cardiac actin (ACTC)***	***FLT1***		***Myocardin (MYOCD)***			
***MLC-2v (MYL2)***			***SRF***			
***Troponin T (TNNT2)***			***TEAD1***			
***Connexin 40 (GJA5)***						
***Connexin 43 (GJA1)***						
***TBX20***						
***MYL4***						
***MYL7***						
***SERCA2A***						

**Fig 4:**
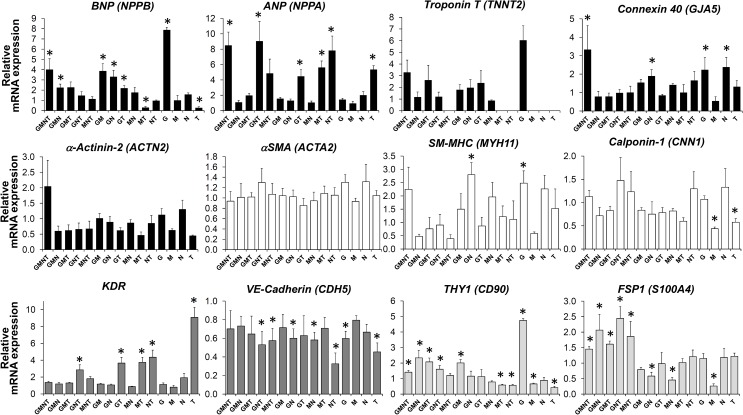
Co-expression of transcription factors in combination in human CPCs. Different combinations of transcription factors (*Gata4* [G], *MEF2C* [M], *NKX2*.*5* [N], and *TBX5* [T]) were introduced to c-kit+ human CPCs via lentivirus. Cells were then cultured for 10 days and analyzed for relative changes in mRNA levels of the indicated genes using quantitative RT-PCR. Cells expressing mCherry served as a negative control (not shown). The level of indicated transcript in each group was compared to that of mCherry control group, and is expressed as a relative fold change. For each condition, *n* = 4. Bar graphs show mean ± SEM. *, *p* < 0.05.

Introduction of *TBX5* also induced significant increases in two cardiomyocyte genes, such as atrial natriuretic peptide (ANP; *NPPA*) and troponin T (*TNNT2*) (Fig 2). However, similar to *Gata4*, *TBX5* failed to induce the full range of cardiomyocyte markers, including α- and β-MHC and BNP (which is closely related to ANP in regard to function and gene regulation), by 2 weeks (data not shown and Fig 2). In fact, *TBX5* overexpression led to suppression of BNP expression, sharply contrasting the effects of *Gata4* (Fig 2). One of the markers that was transiently upregulated by *TBX5* was *KDR* which encodes a VEGF receptor (VEGFR2) (Fig 2). However, it was not accompanied by upregulation of other endothelial cell markers, including VE-Cadherin, CD31, and vWF (Fig 2 and data not shown), and upregulation of KDR alone by TBX5 does not appear to suggest endothelial cell differentiation. With respect to the smooth muscle cell markers, *TBX5* expression resulted in downregulation of SM22α (*TAGLN*) and αSMA (*ACTA2*) (Fig 2). Unexpectedly, *MEF2C* or *NKX2*.*5* alone did not significantly increase the expression of genes that are associated with cardiac differentiation. Similar to *TBX5*, *NKX2*.*5* expression only resulted in a marginal increase in KDR and connexin 40 (*GJA5*) expression by 2 weeks of expression (Figs 2 and 4). Introduction of *MEF2C* was often associated with decreases in the transcripts of smooth muscle cell markers (e.g., Calponin-1 and SM22α), compared to the control (Figs 2 and 4).

Next, we attempted to verify these findings by measuring protein expression of selected markers using immunofluorescence staining and Western blot analysis. For each assay, cells at 2 weeks post-transduction were analyzed. In *Gata4*-overexpressing CPCs, weak yet clear staining for BNP was observed (Fig 3B). In addition, introduction of *Gata4* led to a significant induction of Thy1/CD90 protein in CPCs, whereas its level of expression in mCherry control cells was minimal (Fig 3B). Surprisingly, αSMA protein was also upregulated by *Gata4* (Fig 3B), which was also confirmed by Western blot analysis (Fig 3C). This was unexpected because the qPCR analysis did not show a significant increase in its mRNA levels even after 2 weeks of *Gata4* overexpression (Fig 2). However, we were unable to detect troponin T, ANP or α-actinin protein expression by Western blot analysis although they were readily detected in whole cell lysate of neonatal rat cardiomyocytes (data not shown), suggesting that the level of induction of these markers by the TFs is minimal.

### Effects of overexpression of TFs in combination

**Fig 5:**
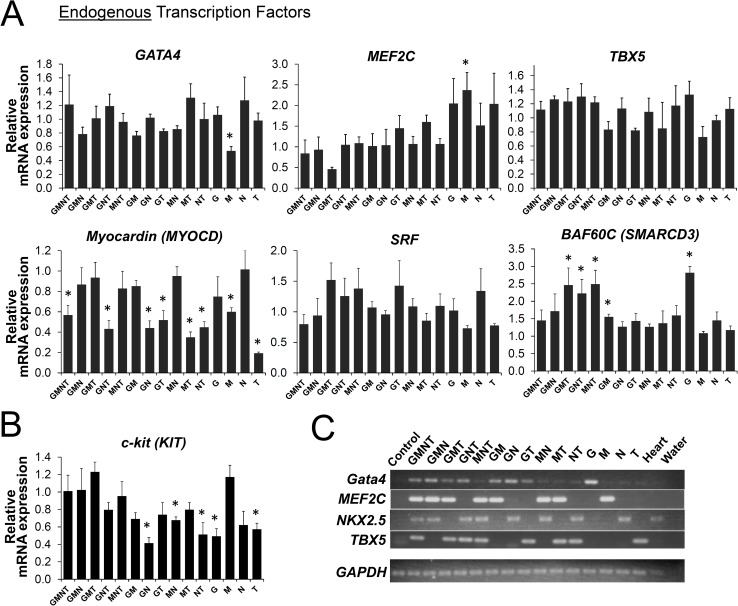
Effect of transcription factor overexpression on the level of endogenous transcription factors. A and B, Different combinations of transcription factors (*Gata4* [G], *MEF2C* [M], *NKX2*.*5* [N], and *TBX5* [T]) were expressed in c-kit+ human CPCs via lentivirus. Cells were then cultured for 10 days and analyzed for relative changes in mRNA levels of indicated genes using quantitative RT-PCR. Cells expressing mCherry (mCh) served as a negative control (not shown). The level of indicated transcript in each group was compared to that of mCherry-expressing control group, and is expressed as a relative fold change. For each condition, *n* = 4. Bar graphs show mean ± SEM. *, *p* < 0.05. C, Representative cDNA sample from each group was analyzed by PCR for each transcription factor to demonstrate the combination of transcription factors overexpressed in each group. PCR products were then analyzed by agarose gel electrophoresis followed by ethidium bromide staining. GAPDH served as an internal control.

Based on these results, we reasoned that a single TF is not sufficient to induce the full range of cardiac marker genes and initiate the cardiac differentiation program in CPCs. Thus, we tested if TFs were more effective in inducing makers of differentiation when used in combination. We tested a total of 15 combinations of four TFs (i.e., Gata4 [G], MEF2C [M], NKX2.5 [N], and TBX5 [T]) (see Fig 4). c-kit+ CPCs were transduced with the combination of viruses as indicated, cultured for 10 days, and analyzed for changes in mRNA expression of differentiation markers by quantitative RT-PCR (see Table 2). The level of indicated transcript in each group was compared to that of mCherry control group. Overexpression of the correct set of TFs in each group was confirmed by quantitative as well as semi-quantitative RT-PCR (data not shown and Fig 5C). Surprisingly, additive or synergistic effects of transcription factors were rarely observed, although with respect to expression of ANP (*NPPA*), connexin 40 (*GJA5*) and α-actinin-2 (*ACTN2*), combination of TFs appeared to be somewhat superior to single TFs (Fig 4). Often, TFs in combination were less effective in inducing differentiation markers compared to single TFs. For example, induction of BNP, troponin T, and THY1/CD90 mRNAs by *Gata4* and KDR by *TBX5* was rather attenuated when combined with other TFs (Fig 4). In contrast, in case of ANP expression, addition of *NKX2*.*5* to *TBX5* appeared to produce a slightly additive effect compared to *TBX5* alone (Fig 4).

Consistent with the lack of robust expression of cardiac marker genes, most of the individual or combinations of TFs were not able to significantly upregulate their endogenous counterparts (i.e., *GATA4*, *MEF2C*, *NKX2*.*5* and *TBX5*) or other important cardiac transcription factors (e.g., *SRF* and *MYOCD* [Myocardin]) (Fig 5A), suggesting that the exogenous factors did not potentiate the endogenous cardiac gene expression program in CPCs. Only *Gata4* was able to reproducibly cause increases in the mRNA levels of *BAF60C* and *TEAD1* (Fig 4 and data not shown). In contrast, *MEF2C* and *TBX5* expression resulted in a significant reduction in expression of myocardin gene (*MYOCD*) (Fig 5A); which is considered a master regulator of smooth muscle gene expression [26]. Among all markers examined, the only mRNA significantly upregulated by *MEF2C* was the endogenous *MEF2C* transcript (Fig 5A), suggesting that a positive feedback regulation of *MEF2C* expression may exist. This is consistent with a previous report that the Mef2c gene is a direct transcriptional target of myogenic bHLH and MEF2 proteins during skeletal muscle development [27]. The endogenous and exogenous transcription factor mRNAs differ in their 5’ and 3’ untranslated regions (UTRs) since the TF constructs only contain the coding sequences and do not contain 5’ or 3’ UTRs of the endogenous TF mRNA. Thus, we designed the primers that specifically target UTRs to detect endogenous TF transcripts only.

### Addition of BAF60C

**Fig 6:**
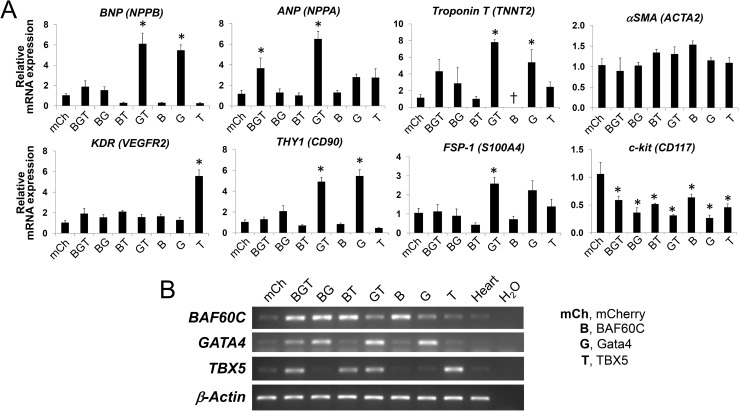
Effect of overexpression of *BAF60C*, *Gata4* and *TBX5* on expression of cardiac differentiation markers in human CPCs. A, Different combinations of three transcription factors (*BAF60C* [B], *Gata4* [G], and *TBX5* [T]) were introduced to c-kit+ human CPCs via lentivirus. Cells were then cultured for 2 weeks and analyzed for relative changes in mRNA levels of indicated genes using quantitative RT-PCR. Cells expressing mCherry (mCh) served as a negative control (not shown). The level of indicated transcript in each group was compared to that of mCherry-expressing control group, and is expressed as a relative fold change. For each condition, *n* = 4. Bar graphs show mean ± SEM. *, *p* < 0.05. B, Semi-quantitative RT-PCR analysis showing increased expression of the indicated transcription factors in each treatment group. β-actin served as a loading control.

Our results suggested that neither individual TFs nor combination of the transcription factors are sufficient to fully program CPCs and suggest that additional factors may be required. One of the candidate proteins to enhance the cardiovascular differentiation of CPCs is BAF60C. The SWI/SNF-like multi-subunit BAF chromatin remodeling complex plays an important role in reorganizing the chromatin structure and facilitating the binding of TFs to their target genes [28]. During the mammalian heart development, Baf60c (Smarcd3), a subunit of the BAF complexes, promotes interactions between transcription factors (e.g., Tbx5, Nkx2.5, and Gata4) and the BAF complex, thereby enhancing transactivation of cardiac genes [29]. In addition, Baf60c was essential for the ectopic cardiogenic activity of Gata4 and Tbx5 in ‘reprogramming’ extra-cardiac mesoderm into heart tissue [18]. We were able to detect mRNA expression of BAF60C in undifferentiated CPCs (Fig 6B), although it was unknown if the protein was present at a sufficient level or if functional BAF complexes were present in CPCs. Thus, we tested the effect of overexpressing *BAF60C* along with *Gata4* and *TBX5* (which were able to induce cardiovascular gene expression in CPCs as shown in Figs 2 and 4). Lentivirus-mediated overexpression of BAF60C was confirmed by Western blot and immunofluorescence staining (Fig 1A and 1B). CPCs were transduced with lentivirus to overexpress either individual or combination of three TFs (i.e., *BAF60C* [B], *Gata4* [G], and *TBX5* [T]). Two weeks later, the cells were analyzed for changes in cardiac gene expression by qPCR. Unexpectedly, *BAF60C* expression did not further enhance expression of cardiac genes induced by Gata4 or TBX5. In fact, in most cases, co-expression of *BAF60C* inhibited the induction of Gata4 and TBX5 target genes in CPCs. For instance, Gata4-induced increases in the level of BNP (*NPPB*), Troponin T (*TNNT2*), THY1/CD90, and FSP-1 (*S100A4*) transcripts by Gata4 were blocked by BAF60C (Fig 6). These observations suggest that BAF60C suppresses cardiac gene expression in CPCs and that increasing expression of BAF60C is not a desired strategy for inducing cardiovascular differentiation of CPCs.

## Discussion

One of the limitations of the current regenerative CPC therapy for ischemic cardiomyopathy is the lack of robust *de novo* differentiation of the transplanted cells in the host myocardium [10, 14]. Although the cause of this is unknown, increasing the cardiogenic differentiation potential of CPCs may further enhance the efficacy of the CPC therapy. In support of this notion, Behfar and colleagues have shown that treatment of cells with ‘cardiogenic cocktail’ prior to transplantation augments the therapeutic benefit of the bone marrow mesenchymal stem cells in chronic ischemic cardiomyopathy [30]. Although previous studies have shown that differentiation of CPCs can be induced by treating the cells with dexamethasone, 5-azacytidine followed by TGF-β1, or co-culturing with neonatal rat myocytes [6, 7, 31–33], the currently available differentiation protocols are often inefficient and result in variable results (Unpublished observations). Thus, in attempt to facilitate differentiation of CPCs, we tested the effects of ectopic expression of five TFs (*Gata4*, *MEF2C*, *NKX2*.*5*, *TBX5*, *and BAF60C*) on ‘priming’ or ‘programming’ CPCs. Such method, often referred to as ‘forward programming,’ has been previously employed to drive cardiomyogenic differentiation of a variety of cell types [18–21, 24, 34], yet has never been tested in CPCs so far. These previous studies have mainly focused on inducing myogenic differentiation of cells of interest, and have not examined the ability of the TFs to induce differentiation of cells into other resident cell types in the heart. In our current study, we conducted a more comprehensive analysis of TF-induced differentiation of CPCs. We assessed the induction of genes associated not only with cardiomyocytes but also with other resident cardiac cell types upon introduction of the TFs. This was based on the observation that CPCs are multipotent and able to give rise to multiple cell types present in the heart, including myocytes, endothelial cells, and smooth muscle cells [6, 7, 9].

**Fig 7:**
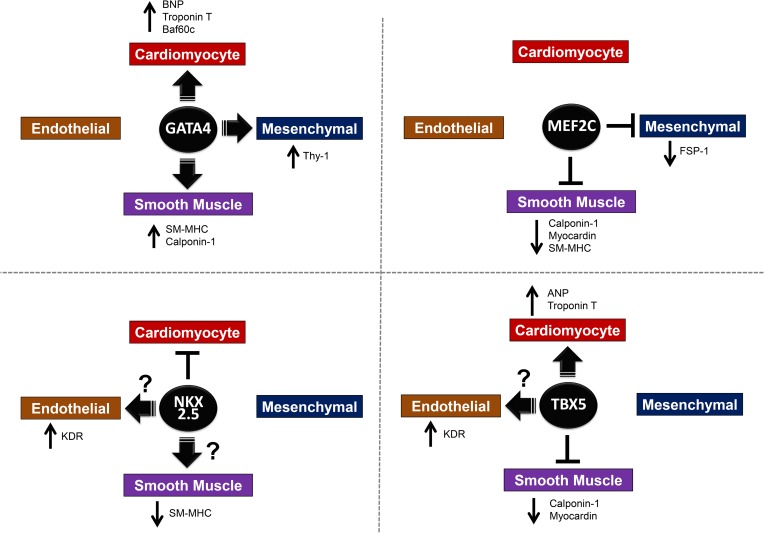
Summary illustration showing the effects that *Gata4*, *MEF2C*, *NKX2*.*5* or *TBX5* overexpression have on cardiovascular differentiation potential of human CPCs. Contribution by TF overexpression to four different cardiovascular lineages (Cardiomyocyte, Endothelial, Smooth Muscle, and Mesenchymal) is shown here based on the gene expression changes induced by each TF. Large arrows indicate promoting action towards the lineage. T-shaped bar indicate inhibitory action towards the lineage. Arrows that are next to genes indicate either increase (upward) or decrease (downward) in the gene expression. Question marks indicate the questionable or inconclusive nature of the association.

Among the TFs tested, *Gata4* seems to be most effective in initiating expression of genes associated with the cardiovascular lineage in CPCs. *Gata4* overexpression was followed by upregulation of markers of multiple cardiac cell types, including myocytes, smooth muscle cells and fibroblasts (Fig 7). Although speculative, this suggests that *Gata4* can facilitate commitment of CPCs down along the differentiation pathway and that ‘priming’ CPCs with *Gata4* can potentially enhance their differentiation characteristics when injected into the infarcted heart. In addition to its role in regulating cardiac gene expression, GATA4 also plays a pro-survival role. Aries *et al*. reported that GATA4 prevents adult cardiomyocyte apoptosis and doxorubicin-induced cardiotoxicity by inducing *Bcl-X*, an anti-apoptotic gene [35]. Also, conditional overexpression of *GATA4* in adult mouse heart increased coronary flow reserve and cardiac contractility by inducing the angiogenic factor, VEGF-A [36]. These findings suggest that enhancing *GATA4* expression in CPCs may provide additional benefits by enhancing their survival as well as promoting angiogenesis in the infarcted myocardium. It should be noted that induction of pro-fibrotic genes, such as THY1 (CD90) and FSP-1, by Gata4 may produce negative impact on the therapeutic potential of c-kit cardiac cells as recent reports have shown that high THY1 (CD90) expression in cardiosphere-derived cells is correlated with limited differentiation potential and lower therapeutic efficacy against MI [37, 38].

Regulation of cardiac differentiation and heart-specific gene expression program is orchestrated by multiple TFs, including GATA4, NKX2.5, TBX5, MEF2 family, and SRF. This notion is supported by the findings that heart-specific enhancer elements are frequently co–occupied and co–regulated by multiple transcription factors [39–41]. Not only do the TFs tested in the current study independently play essential roles in cardiac development, but they also interact with and co-regulate one another [42–51]. For instance, Durocher and colleagues have shown that NKX2.5 interacts with GATA4 and that the two TFs are able to synergistically activate the synthetic (cardiac-specific) rat *Nppa* promoter even in heterologous cells [45]. Also, TBX5 can physically interact with NKX2.5 to synergistically increase the *Nppa* promoter activity [46]. As such, interplay between multiple trans-acting elements is thought to be important in forming a positive regulatory network and initiating cardiac gene expression program. In our current study, however, most of the combinations of TFs failed to act synergistically in activating cardiac gene expression in human CPCs. Instead, co-expression of more than one TF often resulted in suppression of cardiac gene expression compared to single transcription factor alone (see Fig 4). Only in cases of ANP (*NPPA*), connexin 40 (*GJA5*) and α-actinin2 (*ACTN2*), combination of multiple TFs produced slightly higher mRNA expression. Thus, in light of previous reports discussed above, it is not clear why combination of transcription factors were less effective in inducing cardiac gene expression in CPCs. However, it is worth noting that the aforementioned TFs do not always transactivate the target genes in an additive or synergistic manner. For instance, Linhares et al. reported that NKX2.5 and GATA4 transactivate the minimal connexin 40 (*GJA5*) promoter in an additive fashion, yet co-expression of TBX5 strongly suppressed the promoter activity induced by the combination of NKX2.5 and GATA4 [52]. In contrast, activation of ANP (*NPPA*) promoter by TBX5 was further enhanced by co-expression of NKX2.5 and/or GATA4. Taken together, our results suggest that cooperation or antagonism of cardiac TFs is gene-specific, which further demonstrates the complexity of cardiac gene regulatory network.

Our results indicate that neither individual TFs nor combination of the TFs are sufficient to fully program CPCs and suggest that additional factors may be required. For instance, overexpression of *GATA4*, *NKX2*.*5*, or *MEF2C* can enhance cardiomyogenic differentiation of P19 murine embryonic carcinoma [24, 50], yet they do so at differing efficiencies, indicating that their ability to induce cardiogenesis likely depends on the disposition of additional regulatory factors [50]. One of the candidate proteins to enhance the cardiovascular differentiation of CPCs is BAF60C. During the mammalian heart development, Smarcd3/Baf60c, a subunit of the BAF complexes, promotes interactions between transcription factors (e.g., Tbx5, Nkx2.5, and Gata4) and the BAF complex, thereby enhancing transactivation of cardiac genes [29]. However, in our study, co-expression of BAF60C along with Gata4 and TBX5 in CPCs did not further potentiate cardiac gene expression programs initiated by Gata4 and/or TBX5 (see Fig 6). It is generally considered that commitment of stem or progenitor cells to differentiation requires gene expression changes reinforced by epigenetic modifications of the genome [53–56]. Thus, it is possible that complete and robust differentiation of CPCs may require not only induction of TFs but also concomitant programming at the epigenetic level. It would be interesting to test whether treatment of CPCs with DNA methyltransferase or histone deacetylase inhibitors can augment the cardiac gene expression program induced by transcription factors.

In our study, we have used murine Gata4 whereas the rest of TFs used is of human origin. Based on this, one may argue that the lack of cooperativity between TFs is a result of incompatibility of murine Gata4 with human cardiac TFs. Although this is a possibility, we believe that it is unlikely based on previous reports and our own findings. Throughout the cardiac transcription factor literature, investigators have used constructs from various mammalian species. For instance, Sepulveda et al. [48] and Duroucher et al. [45] have shown that mouse Nkx-2.5 and human GATA4 can mutually co-activate avian cardiac α-actin (αCA) promoter and rat Nppa (ANP) promoter. Also, Hiroi et al. [46] have demonstrated that TBX5 and NKX2.5 of human origin can transactivate rat Nppa (ANP) promoter. These reports indicate that, at least within mammalian systems, transcription factors of different species origins can transactivate their target genes. In addition, in our present study, we did observe some synergism between transcription factors. For instance, in cases of ANP (NPPA), connexin 40 (GJA5) and α-actinin2 (ACTN2), combination of multiple TFs produced slightly higher mRNA expression (See Fig 4), suggesting that TFs used in our study can cooperate, but only in the gene context specific manner.

There is always a possibility that any tag or fusion protein interferes with the protein structure and/or function. However, we believe that this is not likely because, in the case of FLAG-tagged Gata4, we observed robust changes in expression of diverse cardiac genes in CPCs. Of note, when the FLAG tag was developed, its structure was optimized for compatibility with the proteins to which it is attached, in that it is more hydrophilic than other common epitope tags and therefore less likely to denature or inactivate proteins to which it is appended [57, 58]. FLAG epitope tagging has grown to become an essential technology in most molecular biology laboratories around the world. In the decades since its original description, it has been applied to study virtually every disease and condition of mankind, and has been adopted in most fields of biology. Hiroi et al. [46] have successfully used FLAG-tagged TBX5 for their study in investigating its interaction with NKX2.5, further supporting the validity of FLAG-tagged constructs.

One of the unexpected findings of the current study was the inability of NKX2.5 to induce cardiac gene expression in CPCs. NKX2.5 is one of the earliest markers of vertebrate heart development [59, 60] and is essential for cardiac differentiation as well as formation of cardiac structures and functions [61, 62]. Accumulating evidence suggests that NKX2.5 is an important regulator of heart-specific gene expression, and often synergizes with other transcription factors [41, 45, 46, 48]. Surprisingly, however, introduction of *NKX2*.*5* into CPCs did not result in upregulation of genes associated with cardiac differentiation. Only marginal increases in connexin 40 (*GJA5*), and *KDR* transcripts were observed following overexpression of *NKX2*.*5* (Figs 2 and 4). Moreover, *NKX2*.*5* did not appear to synergize with other TFs in transactivating the cardiac genes examined in the present study. It is interesting to note that two independent studies which tested TF-induced ‘reprogramming’ of fibroblasts into cardiomyocytes have noted that co-expression of *Nkx2*.*5* along with other TFs unexpectedly hindered cardiomyogenic reprogramming of cells [21, 34]. Currently, it is not clear which variable(s) is responsible for this. We speculate that 1) temporal regulation as well as developmental stage-specific expression of *NKX2*.*5* may be required for the proper activation of cardiac gene expression program; and 2) the activity of *NKX2*.*5* may depend largely upon the cellular context and the array of other transcriptional regulators. Similarly, during the early stage of cardiogenic differentiation, overexpression of *GATA6* transactivated *NKX2*.*5* significantly and stimulated cardiomyogenic differentiation of P19.CL6 mouse embryonic carcinoma cells. However, ectopic overexpression of *GATA6* in undifferentiated P19.CL6 cells was not sufficient to induce *NKX2*.*5* expression [42], indicating that the differentiation-promoting activity of *GATA6* in P19.CL6 cells requires the presence of other cardiogenic transcriptional regulators.

The current study is not free of limitations. There are possible factors which might explain the inefficiency of cardiac differentiation of CPCs by TFs observed in the present study. First, the c-kit+ cells used in our study were not clonally derived and thus likely represent a heterogeneous mixture of different progenitors as suggested previously [63, 64]. Secondly, the cells were isolated from relatively aged MI patients and may not retain their full growth and differentiation potential. Although these factors may indeed play significant roles in dictating the behaviors of CPCs in response to TF overexpression, the cells used in the present study were isolated and cultured in the same manner described for our previous clinical trial (SCIPIO) and therefore, represent clinically relevant populations. Another limitation of the present study is that each combination of TFs were all introduced at the same time and expressed for the same duration. However, activation of cardiac gene programs in CPCs likely requires temporal regulation of the TFs. For this reason, it would be desired to test different temporal or sequential introduction of these transcription factors based on the temporal patterns of TFs during heart development.

In summary, ectopic overexpression of *Gata4*, *MEF2C*, *NKX2*.*5*, *TBX5*, and *BAF60C* was not sufficient to fully activate the cardiac differentiation program in CPCs. The results of the present study show the general lack of synergism between the transcription factors and the selectivity of target genes each transcription factor induces. This underlines the complexity of interplay between multiple *trans*- and *cis*-acting elements that are required for the regulation of cardiac gene expression program. Although introducing *GATA4* to CPCs appears most promising in terms of ‘priming’ them for differentiation, its utility in the *in vivo* setting is unknown and needs further investigation.
